# Immunisation of pigs with recombinant HEV vaccines does not protect from infection with HEV genotype 3

**DOI:** 10.1016/j.onehlt.2023.100674

**Published:** 2024-01-06

**Authors:** Lisa Dähnert, Elmira Aliabadi, Christine Fast, Isabella Hrabal, Charlotte Schröder, Patrick Behrendt, Ulrike Protzer, Martin H. Groschup, Martin Eiden

**Affiliations:** a Institute of Novel and Emerging Infectious Diseases, Friedrich-Loeffler-Institut, Greifswald - Isle of Riems, Germany; b TWINCORE, Centre for Experimental and Clinical Infection Research GmbH, Hannover, Germany; c Helmholtz Centre for Infection Research (Helmholtz-Zentrum für Infektionsforschung GmbH), Braunschweig, Germany; d Department of Gastroenterology, Hepatology and Endocrinology, Hannover Medical School, Hannover, Germany; e Department of Experimental Animal Facilities and Biorisk Management, Friedrich-Loeffler-Institut, Südufer 10, Greifswald-Insel Riems 17493, Germany; f Institute of Virology, School of Medicine, Technical University of Munich / Helmholtz Munich, Munich, Germany; g Partner Site Hamburg-Lübeck-Borstel-Riems and Munich, German Centre for Infection Research (DZIF), Greifswald-Insel Riems 17493, Germany

**Keywords:** Hepatitis E virus, Genotype 3, Recombinant vaccines, Hecolin, Pig infection model

## Abstract

Hepatitis E virus (HEV) is a major cause of acute viral hepatitis worldwide. Up to now, no approved treatment nor a globally licensed vaccine is available. Several recombinant HEV vaccines have been developed to protect against HEV infection in humans, including the commercially available Hecolin vaccine, which are mainly based on HEV genotype 1. However, the efficacy of these vaccines against other HEV genotypes, especially genotype 3 is unknown. In this study, we evaluated the protective efficacy of Hecolin® and a novel genotype 3-based vaccine p239(gt3) against HEV-3 in a pig infection model. Pigs were divided into three groups: one group was vaccinated with Hecolin®, the second group was vaccinated with p239(gt3), and the control group received no vaccine. All pigs were subsequently challenged with HEV genotype 3 to assess the effectiveness of the vaccines. Although all immunised animals developed a high titer of neutralizing antibodies, the results showed that both vaccine applications could not provide complete protection against HEV (gt3) infection: Two out of four animals of the Hecolin® group displayed even virus shedding, and viral RNA could be detected in bile and/or liver of three out of four animals in both vaccination groups. Only one out of four animals in each group was fully protected. Neither Hecolin® nor the novel p239(gt3) vaccine provided sufficient protection against genotype 3 infection. While Hecolin® only partial protected pigs from HEV shedding, the novel p239(gt3) vaccine was at least able to prevent infected pigs from virus shedding. The results highlight the need for further development of HEV vaccines that exhibit broad protection against multiple HEV genotypes and the use of appropriate animal infection models.

## 1 Introduction

Hepatitis E virus (HEV) infections in humans usually lead to an acute, often self-limiting disease, but also cases of fulminant liver failure and extrahepatic manifestation have been reported [1]. According to the World Health Organization (WHO), about 20 million HEV infections are estimated worldwide, leading to about 3.4 million symptomatic cases, 70,000 deaths and 3000 stillbirths annually [2]. HEV consists of a single stranded RNA genome about 7.2 kbp in positive sense polarity which is composed of three open reading frames (ORF). ORF1 includes a methyltransferase, RNA helicase, and RNA-dependent RNA polymerase as well as additional domains with unknown functions. ORF2 encodes for the capsid protein and ORF3 or small phosphoprotein which constitutes a functional ion channel. HEV exists in two states: non enveloped HEV particles were found in feces and bile, while quasi-enveloped particles circulating in blood are completely covered with a lipid membrane, similar to enveloped viruses [3]. Especially during pregnancy, infections with HEV genotypes 1 and 2 are associated with a high risk for fulminant hepatic failure [4]. Due to fecal-oral transmission, large outbreaks have been reported in Africa and Asia caused by contaminated water [5]. Water borne outbreaks are caused by genotypes 1 and 2 (HEV-1/2) that belong to the newly designated subfamily *orthohepevirinae,* genus *Paslahepevirus*, species *Paslahepevirus balayani* [6] and are exclusively restricted to humans. Contrary, the zoonotic genotypes HEV-3 and 4 from the same species are responsible for food borne associated local outbreaks in Europe and Northern America, mainly by ingestion of raw or undercooked meat of infected animals [7]. In addition, an increasing number of transfusion-associated HEV infections through genotype 3 have been notified throughout the world [8]. Moreover, chronic infections can occur in immunosuppressed patients and patients with co-infections or with underlying liver disease [9]. The main natural reservoirs for genotypes 3 and 4 are pigs and wild boar, and to a lesser extent, deer and rabbits [10]. Rabbit-derived HEV forms a clade within genotype 3 of the species *Paslahepevirus balayani* and can infect humans with a pathogenesis similar to that of other HEV-3 subtypes [11]. As a further human pathogenic virus, rat HEV has recently been identified that belongs to the species *Rocahepevirus ratti* of genotype C1 and can infect and is pathogenic for humans [12]. A safe and efficacious vaccine is therefore of particular public-health importance to limit outbreaks and to reduce the transmission of this disease. In particular, the severe courses of the infections caused by HEV-1 and 2 genotypes and the high mortality of up to 30% during pregnancy highlights the need for vaccination of this group of affected individuals. Although in industrialized countries, infection with hepatitis E displays typically mild courses, there is a particular risk of liver failure in patients with advanced liver fibrosis and cirrhosis [13]. Regarding the development of vaccines, there are numerous challenges mainly due to differences between genotypes in terms of transmission modes, distribution, and risk groups. Moreover, relevant aspects such as the circulation of “quasi-enveloped” HEV in blood, the replication in different organs besides the liver and impaired immune responses in vulnerable groups still need clarification [14]. In Europe, no vaccine against HEV is currently licensed, while in China with HEV-239 (Hecolin®, Xiamen Innovax Biotech Co., Xiamen, China) a vaccine has been available for 10 years, and is reported to be highly effective in reducing the risk of HEV-1 and HEV-4 related symptomatic acute hepatitis E [15,16]. However, WHO does not recommend the routine use of the vaccine due to lack of information in children aged <16 years, pregnant women, patients with chronic liver disease, and patients waiting for organ transplantation [17]. Since this HEV-239 (Hecolin®) demonstrated protection against HEV genotypes HEV-1, rabbit HEV and HEV-4 we evaluated the efficiency of the vaccine against swine derived genotype HEV-3 infection. This was performed in an established standard pig infection model with proven exceptionally high susceptibility to HEV-3 [18,19]. In addition, a genotype 3 based p239 vaccine variant, homologous to the challenge strain, was designed and evaluated in the study.

## 2 Material and methods

### 2.1 Experimental design

For this study 10 young domestic pigs (Large White breed) from a commercial breeder (animal husbandry, Dummerstorf, Germany) were acquired and housed under containment level 3** conditions. These animals of compatible sizes and ages were allocated randomly to groups by animal keepers. Social incompatibilities were taken into account in few instances for animal welfare reasons.

The competent authority of the Federal State of Mecklenburg Western-Pomerania has approved all described animal experiments based on European Directive 2010/63/EU and associated national regulation (reference number in Germany LALLF M-V/TSD/7221.3–1.1-022/13).

### 2.2 Inoculum

HEV positive liver tissue of an individual pig from a previous infection study with a HEV-3 (subtype 3i) strain [18] was homogenised and diluted 1:5 with sterile 1× phosphate buffered saline (1xPBS) to obtain a 20% dilution that was subsequently centrifuged at 4400*g* for 15 min at 4 °C. The supernatant was stored at −70 °C until the start of the inoculation.

### 2.3 Vaccine

**Supplemental Fig. S1:**
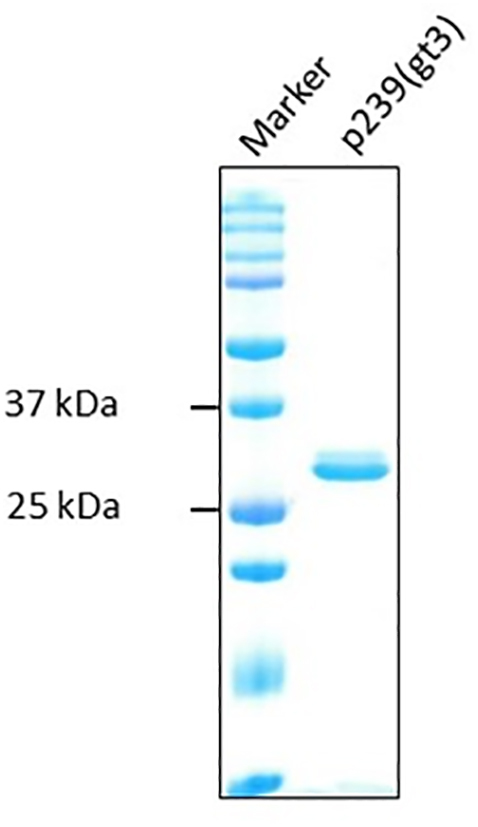
SDS–PAGE analysis and coomassie staining of the bacterially expressed p239 (gt3) protein.

Hecolin (20,149,491, 24-04-2017) was developed by Xiamen Innovax Biotech Co., ltd, China. Hecolin® consists of a 239 amino acid peptide which encodes the partial sequence of a genotype 1 capsid protein [16]. The genotype 3 derived vaccine, known as “Riems vaccine”, is based on the HEV 3i isolate (accession number: KP294371.1) that was also used for infection experiments. The Riems vaccine consists of 239 amino acids of the ORF2 capsid protein (corresponding to nucleotide position 6300–7016 of KP294371). The codon-optimized sequence was synthesized by MWG/Eurofins and cloned into the vector pET19b which harbors a N-terminal His-tag (Novagen/ThermoFisher). Expression in *E. coli* and purification of recombinant protein, using Ni-NTA columns (Qiagen, Hilden, Germany) under denaturing conditions was performed according to the manufacturer's instructions and have been described previously [20]. The elution fractions of the protein were dialyzed against 0.05 M carbonate-bicarbonate buffer pH 9.6 and then stored at −20 °C until use. The corresponding protein is depicted on a Coomassie gel (Fig. S1).

### 2.4 Vaccination and infection procedure

30 μg of the lyophilized Riems vaccine was solved in 0.25 ml NaCl (0,89%.), mixed 1:1 with similar volume AlumVax (containing 0,8 mg Aluminium hydroxide), was injected intramuscularly (i.m.) into 4 pigs followed by a booster shot 28 days later. Similarly, Hecolin® was injected intramuscularly into 4 pigs. As a control, two animals were treated i.m. with pure NaCl solution mixed with a similar volume AlumVax. Four weeks later (56 days post vaccination and 28 days post booster shot), the animals were challenged with a HEV positive liver homogenate from experimentally infected pig (Daehnert et al. 2018). Each animal was infected intravenously (i.v.) with 2.0 ml liver homogenate into vena cava cranialis (Copy number: 2.327 copies/μl RNA corresponding to 8,3 × 10^5^ copies /dose). Serum and feces samples were collected regularly and 15 days post infection (dpi), animals were necropsied and subjected to histological and immunohistological analysis. A summary is depicted in Table 1.

**Table 1:** Vaccination and infection scheme.

	Group 1	Group 2	Control group
Animal number	4	4	2
Antigen	Hecolin®	p239(gt3)	control
Adjuvants	Aluminium hydroxide Al(OH)_3_		
Solvant	0,5 ml buffered saline		
Application	i.m.		
Vaccination	day 0		
Boost	28 day post vaccination		
Challenge	i.v 56 days post vaccination		
Inoculum	HEV homogenate		

Legend: i.m. intramuscularly; i.v. intravenous.

### 2.5 Sampling

Blood/Serum feces samples were collected at regular intervals, usually every 3 or 4-day, and stored at −20 °C. From the feces samples, a 10% (*w*/*v*) fecal suspension was prepared with 0.89% NaCl solution followed by mixing and centrifugation. The supernatant was filtered by a 0.22 μm MILLEX-GP syringe filter unit (Millipore, Ireland) and stored at −20 °C.

### 2.6 Molecular analysis

RNA isolation was performed with 140 μl serum or fecal filtrates, with the QIAmp® Viral RNA Mini Kit (QIAGEN GmbH, Hilden Germany) according to the manufacturer's instructions. RNA extraction from tissue samples was performed with the RNEasy® Mini Kits (QIAGEN GmbH, Hilden Germany) prepared in each case from 10 mg tissue. HEV-derived viral RNA was determined using a quantitative real-time RT-PCR (qRT-PCR) assay targeting a conserved region within ORF3 [21]. The quantification of viral RNA was performed using a synthetic RNA-calibrator comprising the 81 bp target region of the qRT-PCR [21]. MS2-bacteriophage was added and used as RNA extraction control [22].

### 2.7 Serological analysis

The immune response was tested using serum samples with the commercially available species independent HEV-Ab ELISA (AXIOM, Bürstadt, Germany), which detects total serum anti-HEV-antibodies.

### 2.8 Neutralization assay

Naked HEV genotype 3 viral strain Kernow C1p6 G1634R was produced as described previously [23]. In brief, serum samples were titrated in duplicate in five threefold serial dilutions (starting from a dilution 1:300) in MEM low IgG FCS medium. Each serum dilution (40 μl) and medium (40 μl) containing naked HEV genotype 3 viral strain Kernow C1p6 G1634R were mixed and incubated at 37 °C for 1 h. As negative controls, parallel assays were run with pig sera prior to vaccination. Subsequently, the serum-virus mixtures were added to human hepatoma cell line HepG2/C3A cells seeded in 96-well plates and incubated at 37 °C and 5% CO_2_. After 24 h, 100 μl fresh medium was added. Four days poste infection, cells were fixed with 3% paraformaldehyde (PFA) in PBS, permeabilized and stained for the ORF2-encoded capsid protein. The number of focus-forming units (FFUs) were counted using ELISpot reader. The endpoint FFU in the presence of pig serum was calculated through the following formula: 100 × [average FFU in the presence of serum from the vaccinated animal / average FFU in the in the presence of serum from either the control animals or the vaccinated animals prior to the time point of vaccination].

### 2.9 Gross examination, histopathology and immunohistochemistry

All animals were examined post-mortem for gross pathological lesions. Randomly distributed samples were taken from each liver globe, gall bladder and hepatic lymph node were taken and immediately fixed in 10% buffered formalin (4% solution of formaldehyde). After dehydration all tissue samples were embedded in paraffin and 3 μm sections were prepared, rehydrated and stained with hematoxylin and eosin (H&E) for histopathological examination. For immunohistochemistry rehydrated 3 μm sections were pretreated by blocking the endogenous peroxidase activity with 0.3% hydrogen peroxide in methanol for 30 min, by incubating with TRIS/EDTA buffer (pH 9) at 121 °C for 20 min and by blocking unspecific reactions with 1:1 diluted goat serum in TBS for 10 min at room temperature. Immediately after blocking sections were incubated for 2 h at room temperature with FLI in-house monoclonal antibody 6A2 (1:25) respectively in-house polyclonal antibody JS443 (1:600), both diluted in goat serum (10% diluted in TBS). Monoclonal antibody 6A2 derived from a hybridoma cell clone that was generated by immunisation of BALB/c mice with recombinant p239 protein using standard hybridoma techniques. Negative control sections were incubated with goat serum alone. Slides were finally developed with EnVision reagent (Dako Diagnostics, Hamburg, Germany) and diaminobenzidine tetrahydrochloride, counterstained with Mayer's hematoxylin.

## 3 Results

**Supplemental Fig. S2:**
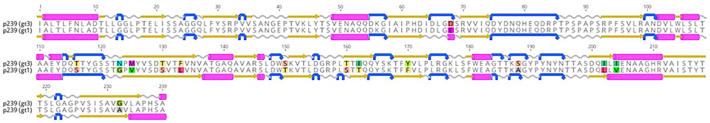
Secondary structure and alignment of p239 (gt3) with a genotype 1 reference strain from China (accession number L08816). Differing amino acids are color-coded.

A homologous prime-boost strategy was used by immunisation of pigs with two different non-glycosylated recombinant antigens: Hecolin® and p239(gt3). The Hecolin sequence is based on a Chinese human HEV genotype 1 strain in contrast to p239(gt3), which is derived from a German wild boar HEV genotype 3 isolate. Both encompass a 239 amino acid of the viral capsid protein located at amino acid position 368–606. The p239 (gt3) vaccine has a molecular weight of about 25.6 kDa (supplemental Fig. S1) and exhibits 14 amino acid exchanges compared to a genotype 1 reference strain. The alignment is depicted in supplemental Fig. S2.

**Fig. 1:**
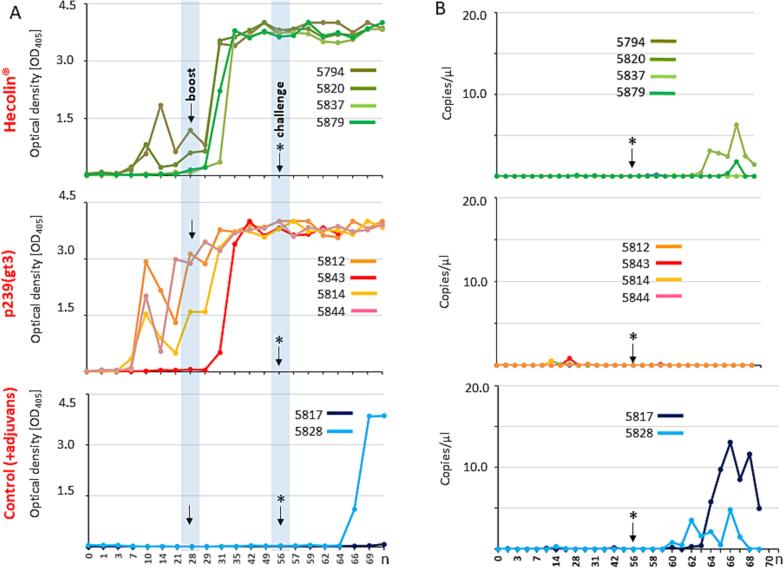
Antibody development after vaccination starting with initial immunisation, boost at day 28 and challenge at day 56. Figure displays the individual curves of antibody titer obtained from ELISA analysis (A). Results of RT-qPCR of shed HEV RNA from fecal samples over the course of vaccination and challenge (B). Arrow indicates date of boost. Arrow with star indicates date of infection. n: necropsy.

Vaccination was performed by intramuscular (i.m.) injection the antigens and 28 days later with the same boost regimen. A summary of vaccination outcome is seen in Fig. 1 including course of antibodies (Fig. 1 A) and viral RNA shedding (Fig. 1B). After immunisation with Hecolin two out of 4 animals developed antibodies against HEV starting from day 7 on. The booster administered 28 days post vaccination (dpv) induced antibodies against HEV in all four animals leading to a high and stable antibody titer at 35 dpv which was maintained until the end of the experiment. Similarly, vaccination with p239 (gt3) induced antibodies against HEV in three out of four animals. After the booster was administered 28dpv, a stable antibody titer formed for all animals from day 35 until necropsy. The unvaccinated control group, in which only the adjuvant was administered alone, did not develop antibodies during the prime-boost regimen. Following the immunisation, at day 56 animals were infected by intravenous inoculation of a HEV positive liver homogenate.

Both non-vaccinated control animals (nr 5817 and nr 5828) displayed virus shedding starting on day 60 and 64 (Fig. 1B), respectively. In addition, pig 5828 also exhibited seroconversion from day 66 onwards. This increase in antibody titer was accompanied by a reduced virus shedding. In contrast to animal 5817 revealed no seroconversion but showed an increased amount of viral shedding in feces.

Within the Hecolin® vaccination group, two out of four animals displayed shedding of viral RNA that was observed shortly after infection at days 6 and 8 post infection (dpi), which corresponded to 62 and 64 days post vaccination, respectively. In contrast, viral RNA was not detected in the feces of any of the 4 animals in the p239(gt3) vaccination group. Finally, viral.

viral RNA could not be detected in any serum samples from the animals.

**Fig. 2:**
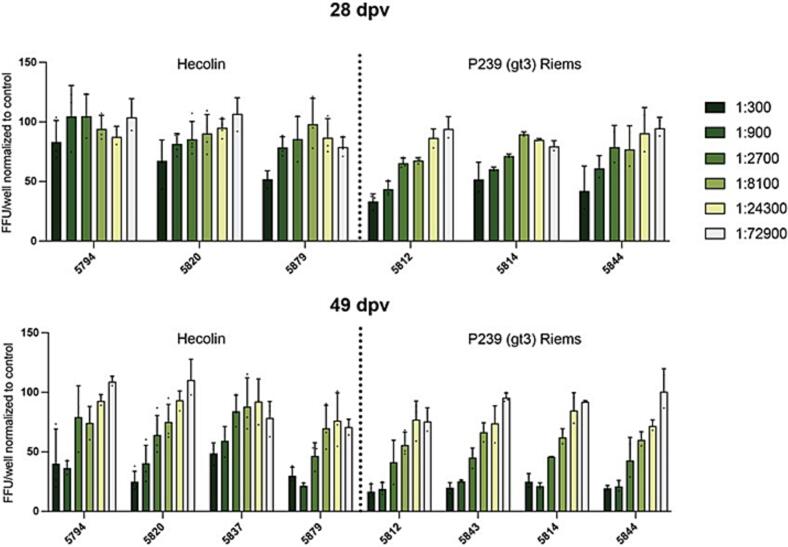
Neutralization activity of pig sera against naked form (*N* = 2–4) of genotype 3 viral strain Kernow C1p6 G1634R on day 28 and day 49 post vaccination. Focus forming units (FFU) were counted after incubation of the virus with sequential sera from pig immunised with Hecolin or p239 (gt3). Results are normalized to serum samples before vaccination. Sera of animals 5837 (28dpv) and 5843 (28dpv) were no longer available.

In order to determine neutralizing activity of pig serum following immunisation and subsequent boosting, corresponding samples collected from day 28 and day 49 post-vaccination were subjected to a neutralization assay based on HEV genotype 3. Our results showed that the neutralization capacity of serum against HEV-3 was higher at day 49 compared with day 28 post-vaccination, attributable to the effect of the boosting process (Fig. 2). Moreover, the neutralization activity of serum from animals vaccinated with vaccine p239 (gt3) was slightly stronger than that from animals vaccinated with Hecolin on both day 28 and day 49 post-vaccination. These findings suggest that p239 (gt3) may confer slightly higher neutralization activity compared to Hecolin.

At 71 days post vaccination and 15 days post infection, all animals were necropsied and different tissues/organs were subjected to molecular and histopathological/ immunohistochemical analysis.

Two out of four animals from the Hecolin© vaccination group (2/4) were positive for HEV RNA in the liver and bile (animals 5837 and 5879). Positive bile samples were also observed in three out of four animals (5843, 5814, 5844) in the p239(gt3) vaccination group. One of the animals (5844) was also positive in the liver. Both non-vaccinated animals were positive either in the bile (5828) or in the liver (5817). A compilation of the results from molecular analysis is shown in Table 2.

**Table 2:** Results of RT-qPCR analysis of selected tissue samples.

Vaccine	Animal	Tissue				
		Bile	Liver 1*	Liver 2*	Liver 3*	Liver 4*
Hecolin	5794	128.4	neg	neg	neg	neg
	5820	neg	neg	neg	neg	neg
	5837	167.19	1.74	0.8	4.45	7.61
	5879	31.07	neg	4.49	neg	neg
p239 (gt3)	5812	neg	neg	neg	neg	neg
	5843	83.06	neg	neg	neg	neg
	5814	11.77	neg	neg	neg	neg
	5844	125.05	neg	neg	35.94	neg
control	5817	neg	6.54	13.98	6.05	9.85
	5828	1133.58	neg	2.79	neg	neg

Legend: PCR results are indicated in cop/μl RNA. * Tissue samples were randomly taken from each liver lobe.

**Fig. 3:**
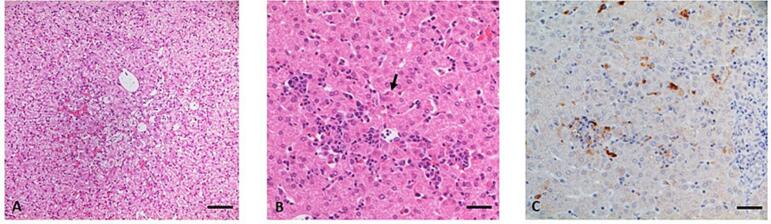
Histopathological and immunohistochemical examination of the liver. (A) mild centrobular hydropic degeneration of hepatocytes and mild congestion of hepatoid sinusoids, pig #5843, H&E, bar 50 μm; (B) mild intralobular lymphohistiocytic infiltration and degeneration of single hepatic cell (arrow), pig #5837, H&E, bar 20 μm; (C) immunohistochemical detection of intracellular HEV antigen mainly in Kupffer and infiltrating inflammatory cells, pig #5837, immunohistochemistry, mab 6A2 (FLI in house antibody), bar 25 μm.

In histopathology (Table 3) multifocal acute mild (5843, 5844, 5817) to moderate (5837) lymphohistiocytic hepatitis was seen in four animals (4/10) from all groups. These alterations were characterized by randomly distributed intralobular necrosis of individual hepatocytes (3/4) accompanied by mild up to moderate lymphohistiocytic infiltrates (4/4) and in some animals also by mild acute hemorrhages (3/4). Very slight indications of such alterations were also noted in the remaining six animals (6/10). One of the pigs (5843) showed, not only hepatitis but also an oligofocal acute centrolobular hydropic degeneration of hepatocytes. In eight animals from all groups (8/10) a multifocal mild periportal lymphohistiocytic (1/8), lymphoplasmacytic (7/8) or eosinophilic infiltration was detectable. Additionally in five animals (5/10) a mild to moderate bile duct proliferation was seen. Furthermore, one animal showed a moderate follicular hyperplasia in the hepatic lymph node. Intracellular viral antigen was detectable in only one animal (5837) focally associated with Kupffer and infiltrating mononuclear inflammatory cells (histiocytes, lymphocytes) of the liver as well as in individual mononuclear cell of the hepatic lymph node (Fig. 3).

**Table 3:** Histopathological findings in tissues of challenged pig.

Group	Animal ID	Liver	Hepatic lymph node	Gallbladder
Hecolin®	5794	- -	- -	- -
	5820	- -	- -	- -
	5837	Multifocal mild acute non-suppurative hepatitis, mild viral antigen in Kupffer and inflammatory cells	Individual mononuclear cells with viral antigen	- -
	5879	- -	- -	- -
p239(gt3)	5812	- -	- -	- -
	5843	Oligofocal mild acute non-suppurative hepatitis, focal mild hydropic hepatocellular degeneration	- -	- -
	5814	- -	Moderate follicular hyperplasia	- -
	5844	Multifocal mild acute non-suppurative hepatitis	- -	- -
control	5817	Multifocal mild acute non-suppurative hepatitis	- -	- -
	5828	- -	- -	- -

Legend: (**−-**) nothing to report.

## 4 Discussion

A number of protein-based vaccines, all based on genotypes 1, 2 or 4 so far, have been developed against HEV and were evaluated in small animal models as well as primates. In each case, variants of the ORF2 derived capsid protein either recombinant expressed segments and domains, vectored vaccines or combined/chimeric vaccines were used [25].

The only HEV vaccine approved to date is Hecolin(® from Xiamen Innovax Biotech Co, Ltd.in Xiamen, China [26]. It comprises a bacterial expressed 239 amino acid long partial fragment (amino acids 368–606) of a human genotype 1 strain. In animal infections studies, including rabbits and rhesus monkeys, it has demonstrated protective efficacy against HEV-1 and HEV-4 as well as rabbit HEV strains but not against HEV-3 strains [[26], [27], [28]]. In studies involving primates, intramuscular immunisation with either 10 or 20 μg of the p239 vaccine administered at weeks 0 and 4 yielded a robust antibody response. This immunisation schedule conferred complete protection upon intravenous injection of a 104 dose of both HEV-1 and HEV-4 strains. However, in the face of a higher dose of 107, only partial protection was evident. Each experiment comprised groups of three individuals, and protection was evaluated through RT-PCR analysis of fecal excretion [26]. Subsequently, analogous immunisation experiments with Hecolin were carried out using rabbits, also organised in groups of three. Following triple intramuscular immunisation at weeks 0, 2, and 4 with 30 μg of Hecolin each, the rabbits were once again challenged with a HEV-4 and a rabbit HEV strain at a dose of 2.3 × 106 copies/ml. This protocol elicited a robust immune response in all immunised rabbits, with no observable fecal virus shedding as analysed by RT-PCR [27].

In another experiment involving a swine HEV-4 and the same rabbit HEV strain, various immunisation strategies were employed. Notably, it was demonstrated that two immunizations with 10 μg of Hecolin provided complete protection against HEV infection compared to a single administration of 20 μg or 30 μg of the vaccine. This particular study included the analysis of 8 animals per group, and fecal excretion of viral RNA was determined again through RT-PCR assays [28].

There were only cell-based studies on protection against genotype 3 strains showing that infection of HepG2 cells with the genotype 3 Kernow strain was blocked by p239(gt3) vaccinated human serum [29]. The vaccine has been licensed in December 2011 by the China Food and Drug Administration [17] for persons from the age of 16 years with the risk of HEV infection and has been intensively evaluated in Phase III study [15] and a follow-up study [30]. Previously, two phase IV studies were performed where the efficacy of HEV-239 in a modified, accelerated vaccination schedule was tested [31] as well as the safety and tolerability in women of childbearing age in Bangladesh [32]. To our knowledge no evaluation of Hecolin efficacy against infection with zoonotic genotype 3 strains has been conducted. Therefore, we used a well-established pig infection model [18,19,37] to analyze immunogenicity and protectivity of this vaccine against HEV-3 strains. For comparison, a first genotype 3i-based vaccine (p239 Riems), which encompassed the same position within the ORF2 (amino acid 368 to 606), was included in the study.

Immunisation with a prime-boost regimen induced a high antibody titer for both vaccines in all individual pigs and reached a plateau at day 35 post initial vaccination, that was maintained until the end of the experiment. All animals developed antibodies with neutralizing efficiency, which was enhanced again after the boost, against HEV-3 *in vitro*. It was shown that the neutralizing activity against the genotype 3 virus strain Kernow *in vitro* was slightly higher with the p239(gt3) vaccine than with Hecolin, which can be attributed to genotype 1 based sequence of Hecolin. A similar neutralization efficiency *in vitro* has been observed in sera from vaccinated humans [29]. However - *in vivo* - immunisation with Hecolin showed sterilizing protection only in one out of four animals, after the subsequent challenge. In two Hecolin immunised animals, virus shedding was detected in feces samples. Moreover, in necropsied animals, viral RNA was detected in liver of three animals as well as in the bile of two animals. In contrast, none of the p239(gt3) immunised animals excreted viral RNA to feces. However, similar to the Hecolin group, in three out of four animals, virus RNA was detected in the bile as well as the liver of one individual. In the control group, both animals shed virus and exhibited HEV positive tissues either in bile or bile and liver. Only in one animal HEV antigen was detectable in liver and hepatic lymph node. This animal had also not only the most pronounced hepatitis in our study, but viral antigen was also mainly associated intralesionally with Kupffer and infiltrating inflammatory cells in the liver. Moreover, this result agrees well with the results of the molecular analysis. Therefore, we do not consider it unlikely that the observed lesions in other pigs of the study are also related to the HEV infection, although we were unable to detect viral antigen by immunohistochemistry in these animals. This is underlined by previous studies describing similar lesions in HEV infected pigs and wild boars [18,33]. However, negative control animals would be required to definitively exclude another cause for these rather nonspecific liver lesions. Unfortunately, such animals were unfortunately not included in the study.

A similar immunisation study with genotype 3 based p239 vaccine was performed recently, which also demonstrated absence from virus shedding, after infection with a swine derived HEV-3 strain in pigs. However, it was not possible to detect the virus in the tissues because the animals were not dissected until 10 weeks after inoculation [34]. In this study a baculovirus expressed p239 derived virus-like particle (VPL) based on a Korean genotype 3 strain was generated. Immunisation and booster was done at week 0 and week 2 respectively by i.m. application, either with 100 μg or 200 μg of the VLP vaccine, followed by intravenous challenge (106 HEV copies/dose) with a swine HEV-3 strain. Groups of 3 individuals were combined for this purpose. Again, the effectivity of the immunisation was assessed by analysis of viral shedding and analysis of viral RNA by RT-PCR. Only the 200 μg formulation could provide complete protection from virus shedding.

The data demonstrate an ineffective protection - with regard to sterilizing immunity- of both vaccines against HEV infection with swine-derived HEV-3 strain. The ineffectiveness of the Hecolin vaccine is remarkable, since several studies in rabbits demonstrated full protection against HEV-3 [35]. However, in all these cases rabbit derived HEV-3 (rabHEV) strains were used, which displays specific sequence features including a unique 81 nucleotide insertion within ORF1. Infections of rabbits with rabbit derived HEV-3 strains induce fecal shedding, viremia and liver histopathological changes [36]. Contrary, infection of swine or wild boar derived HEV-3 strains in rabbits exhibit low infection efficiency [37,38] or no replication when human derived HEV-3 strain was used [36].

The second point is the exceptionally high susceptibility of pigs to HEV-3, with only 6.5 copies being sufficient for infection [18]. There are no comparable titration studies for rabbits or primates so far, which makes pigs an appropriate model for evaluation of Hepatitis E vaccine efficacy, especially with regard to further human application. Further studies on human and swine-derived HEV-3 but also of HEV-4 strains should be performed in order to determine the protective efficacy of established as well as novel vaccine candidates.

The following are the supplementary data related to this article.

## CRediT authorship contribution statement

**Lisa Dähnert:** Formal analysis, Methodology, Resources, Validation, Writing – original draft, Writing – review & editing. **Elmira Aliabadi:** Data curation, Investigation, Methodology, Resources. **Christine Fast:** Data curation, Investigation, Methodology, Resources, Writing – review & editing. **Isabella Hrabal:** Formal analysis, Methodology, Resources. **Charlotte Schröder:** Investigation, Methodology, Resources. **Patrick Behrendt:** Data curation, Formal analysis, Investigation, Methodology, Resources. **Ulrike Protzer:** Conceptualization, Data curation, Formal analysis, Methodology, Resources, Writing – review & editing. **Martin H. Groschup:** Conceptualization, Formal analysis, Methodology, Supervision, Validation, Writing – review & editing. **Martin Eiden:** Conceptualization, Data curation, Methodology, Supervision, Validation, Writing – original draft, Writing – review & editing.

## Declaration of competing interest

The authors declare that they have no known competing financial interests or personal relationships that could have appeared to influence the work reported in this paper.

## Data Availability

Data will be made available on request.
